# The Evolutionary Dynamics of a Rapidly Mutating Virus within and between Hosts: The Case of Hepatitis C Virus

**DOI:** 10.1371/journal.pcbi.1000565

**Published:** 2009-11-13

**Authors:** Fabio Luciani, Samuel Alizon

**Affiliations:** 1 Centre for Infection and Inflammation Research (CIIR), School of Medical Sciences, University of New South Wales, Sydney, Australia; 2 Institut für Integrative Biologie, ETH, Zürich, Switzerland; Imperial College London, United Kingdom

## Abstract

Many pathogens associated with chronic infections evolve so rapidly that strains found late in an infection have little in common with the initial strain. This raises questions at different levels of analysis because rapid within-host evolution affects the course of an infection, but it can also affect the possibility for natural selection to act at the between-host level. We present a nested approach that incorporates within-host evolutionary dynamics of a rapidly mutating virus (hepatitis C virus) targeted by a cellular cross-reactive immune response, into an epidemiological perspective. The viral trait we follow is the replication rate of the strain initiating the infection. We find that, even for rapidly evolving viruses, the replication rate of the initial strain has a strong effect on the fitness of an infection. Moreover, infections caused by slowly replicating viruses have the highest infection fitness (i.e., lead to more secondary infections), but strains with higher replication rates tend to dominate within a host in the long-term. We also study the effect of cross-reactive immunity and viral mutation rate on infection life history traits. For instance, because of the stochastic nature of our approach, we can identify factors affecting the outcome of the infection (acute or chronic infections). Finally, we show that anti-viral treatments modify the value of the optimal initial replication rate and that the timing of the treatment administration can have public health consequences due to within-host evolution. Our results support the idea that natural selection can act on the replication rate of rapidly evolving viruses at the between-host level. It also provides a mechanistic description of within-host constraints, such as cross-reactive immunity, and shows how these constraints affect the infection fitness. This model raises questions that can be tested experimentally and underlines the necessity to consider the evolution of quantitative traits to understand the outcome and the fitness of an infection.

## Author Summary

Rapidly mutating viruses, such as hepatitis C virus, can escape host immunity by generating new strains that avoid the immune system. Existing data support the idea that such within-host evolution affects the outcome of the infection. Few theoretical models address this question and most follow viral diversity or qualitative traits, such as drug resistance. Here, we study the evolution of two virus quantitative traits—the replication rate and the ability to be recognised by the immune response—during an infection. We develop an epidemiological framework where transmission events are driven by within-host dynamics. We find that the replication rate of the virus that initially infects the host has a strong influence on the epidemiological success of the disease. Furthermore, we show that the cross-reactive immune response is key to determining the outcome of the infection (acute or chronic). Finally, we show that the timing of the start of an anti-viral treatment has a strong effect on viral evolution, which impacts the efficiency of the treatment. Our analysis suggests a new mechanism to explain infection outcomes and proposes testable predictions that can drive future experimental approaches.

## Introduction

Rapidly mutating viruses, such as hepatitis C (HCV) and Human immunodeficiency virus (HIV), are very successful at surviving in their hosts because of their rapid evolutionary dynamics, which allow them to evade the immune response. Models have been developed in evolutionary epidemiology that link within- and between-host dynamics [1]–[8]. One of the main results of these so-called ‘nested models’ is to show that intermediate replication rates maximise the fitness of an infection (which is known in epidemiology as the 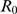 and corresponds to the number of new infections caused by an infected host in a susceptible population [9]). However, none of these models take into account within-host evolution. Here, we allow for the replication rate to evolve during the course of an infection and derive the invasion fitness of a rapidly mutating pathogen. In this study, we focus on the case of HCV.

### Modelling within-host evolution

Most theoretical and experimental studies focus on the dynamics of viral diversity during an infection (see e.g., [10]–[14]) or on qualitative traits such as drug resistance [15]–[17]. Studies rarely consider quantitative traits, even though viral replication seems to provide an exception. Variable polymerase activities have been observed during HCV infections along with the individual viral sub-populations, both between infected individuals and within an individual [18], which suggests that strain distribution in a given host is characterised by a range of replication rates. Given the high evolutionary rates of rapidly mutating viruses [19],[20] and the extreme selection pressure exerted by the immune response [15],[21], one can expect such quantitative traits to evolve during an infection.

Infections caused by a rapidly mutating virus exhibit high levels of genetic diversity. Classical theory predicts that, in such a case, faster replicating strains have the highest infection fitness because they gather more resources before the end of the infection [22],[23], which has been confirmed experimentally (e.g., [24]). This ‘short-sighted’ evolution, with the subsequent selection of faster replicating strains, has been proposed as a mechanism to explain HIV virulence [23]. Yet, not all strains have high replication rates and rapidly replicating strains also kill their host quickly, thus potentially decreasing their fitness at the between-host level [25]–[27].

Some within-host models study the evolutionary dynamics of the replication rate during the course of an infection. A common finding of such models is that the replication rate increases during the course of an infection, either because of resource competition or because of apparent competition through the immune system [28]–[30]. If within-host trade-offs are assumed, strains with intermediate or low replication rates can take over the infection [31]–[35].

Here, we focus on the fitness of a viral strategy at the between-host level (i.e., how many new infections an infection can cause). Bonhoeffer and Nowak [25] developed one of the few evolutionary epidemiology models that includes within-host evolution of the replication rate, however they simplified within-host processes to the extreme by assuming that, within a host, a more virulent mutant always instantaneously takes over the infection. Our model describes the within-host dynamics, which allows us to follow the evolution of the virus along with the changes in its environment (i.e., the immune response).

### Immune activation as a constraint

The immune system is known to be a major constraint on viral evolution [21],[36]. This underlines the importance of including immune dynamics in within-host models [37],[38]. Following [7], we assume that immune activation depends on the overall viral growth rate, i.e., the product of the viral replication rate and density. The idea underlying this assumption is that increasing the growth rate can be detrimental for the virus because it increases immune pressure. An implication of such a trade-off is that a strain with a lower growth rate can have higher infection fitness because the infection lasts longer.

This assumption echoes the immunogenicity criterion formalised by Pradeu and Carosella [39]: maintaining a *continuity*, in terms of antigens (viral peptides recognised by the immune system) present in the host, can be decisive for the pathogen to avoid triggering an immune response. How can the replication rate affect this continuity? Infected cells process and present antigenic peptides, that are ‘pictures’ of the current intracellular state, to the initial T-cell repertoire. Yewdell [40] argues that there is a clear link between viral translation (i.e., the production of non-endogenous proteins) and antigen presentation. The reason for this is that rapidly degraded polypeptides (associated with rapid translation) lead to a more efficient generation of major histocompatibility complex (MHC) Class-I peptides, which are then presented to cytotoxic T cells. This explains why infected cells can be identified so rapidly: viral peptides have a higher chance to be expressed than endogenous peptides because they are degraded more rapidly than endogenous gene products [40]. Rapidly replicating viruses could then be an easy target for the immune system if they generate a larger pool of rapidly degraded polypeptides. This is consistent with the fact that 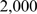 to 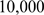 copies of a protein are needed for successful processing and presentation of MHC Class I peptides [41]. A few experimental studies have also shown that increasing the viral replication rate provides a wider and more abundant antigen presentation [40],[42],[43]. One must admit that it is difficult to disentangle the roles of variations in replication rates and variations in viral density because the two are obviously linked [7]. Moreover, many other factors such as the dose, the localisation and the duration of antigen presence are also critical to immune activation [44].

Here, we develop a nested model that links within-host evolutionary dynamics to the epidemiology. By explicitly describing the immune dynamics, the model takes into account the fact that a viral strategy dominant early in an infection (e.g., replicating slowly to escape the immune system) may be rare later in the infection.

## Results

We perform stochastic simulations with a model that describes the within-host evolution of a viral strain undergoing mutation. In our model, the immune response is assumed to be strain specific and we account for cross-reactive immunity, i.e., the fact that strains are recognised by more than one clone of lymphocytes. Viral strains are identified by their replication rate and their antigen value, the latter defining the extent to which the strain is recognised by a given T-cell clone. We introduce between-host dynamics by adding transmission events whose probabilities depend on the viral load at a given time (see the Model section and Table 1 for a description of the variables and parameters used).

**Table 1:** List of the variables and parameters of the model.

Notation	Description	Value
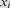	Number of cells infected with viruses of strain 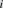	variable
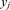	Number of immune cells specific to strains with antigen 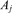	variable
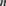	Number of viral strains	variable
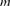	Number of lymphocyte clones	20
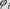	Replication rate of viral strain 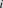 (day^−1^)	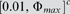
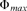	Maximum replication rate (day 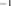 )	4
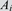	Antigen value of viruses of strain 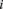	variable
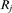	Receptor value of lymphocytes of clone 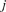	variable
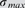	Maximum killing rate (day^−1^ lymphocyte^−1^)	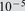
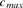	Maximum activation rate of lymphocytes (infected cells^−1^)	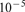
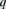	Breadth of immune cells specificity	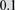
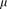	Virus mutation rate (per generation)	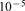
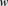	Width of the distribution of replication rates	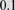
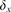	death rate of infected cells (day^−1^)	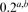
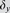	death rate of lymphocytes (day^−1^)	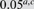
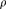	Probability of transmission to another host (per generation)	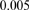
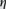	Probability of virulence, i.e. host death (per generation)	0
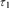	Intensity of a virus-clearance treatment (day^−1^)	0
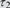	Intensity of a replication-blocking treatment (day^−1^)	0

Ranges for parameter values for the death rates and for the killing rate are taken from a [82], b [51], c [42] and d [83].

**Figure 1:**
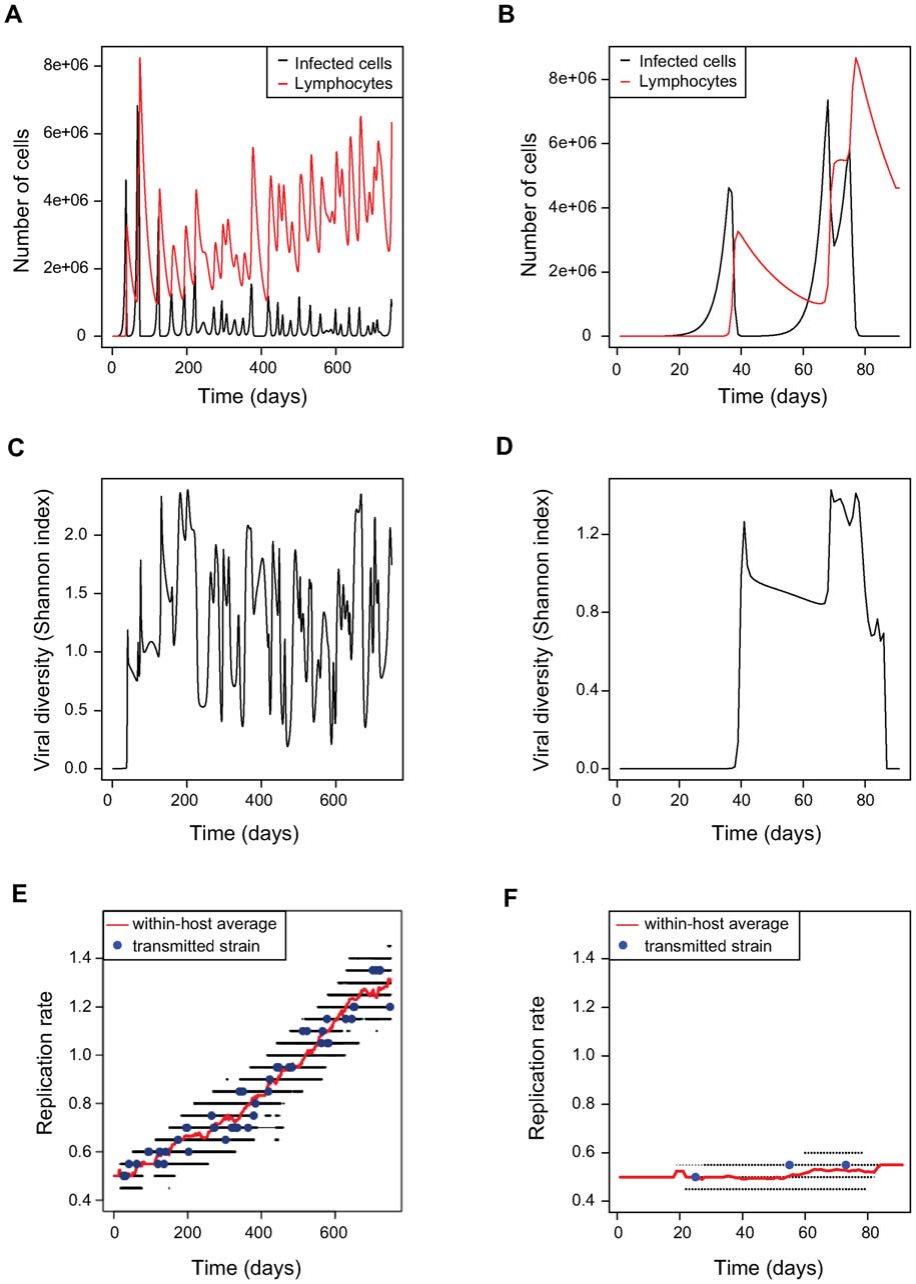
Within-host population dynamics and evolutionary dynamics for two “typical” simulation runs. With identical parameter values, we can observe a chronic (A,C,E) or an acute (B, D, F) infection. A) and B) Population dynamics of infected cells (in black) and immune cells (in red). C) and D) Diversity dynamics (Shannon index). E) and F) Evolutionary dynamics of the replication rate. Black dots indicate the replication rates present at any time, the red line is the average replication rate for each time t, and the large blue dots show the replication rates of the strains that are transmitted to another host after a transmission event. Here 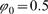. Other parameter values are default and given in Table 1.

Figure 1 shows the outcome of two ‘typical’ stochastic realisations of the within-host evolutionary dynamics performed with default parameter values. The first result is that the exact same parameter values can lead to a chronic (A, C and E) or an acute (B, D and F) infection. Panel A shows that both the viral load and the number of immune cells vary strongly during the early phase of the infection, which is consistent with observations from several chronic infections (e.g., [45]). Also, the increase in the number of immune cells and infected cells occurs two weeks after the beginning of the infection, which is in line with clinical observations [46]. In chronic infections, the overall density of immune cells increases slightly (Figure 1A), which is likely to be linked with the increase in the average virus replication rate (see below). Longitudinal analyses of cellular immune responses to HCV in chimpanzees and humans, although limited to small sample sizes, show that immune responses may persist during the chronic phase and eventually stabilise to levels that depend on host immunity and on the viral strain [47],[48]. Panels C and D show that diversity increases rapidly within a few days. In the case of chronic infections (Figure 1C), diversity reaches a threshold. This mutation-selection equilibrium is constrained by the fact that the antigenic space is finite. Panels E and F show that there is also diversity in viral replication rates and that, for these parameter values, the average replication rate tends to increase during the infection. This increase is a result of the apparent competition generated by the cross-reactive immune response (see also [8]).

Within-host evolutionary dynamics are expected to affect the dynamics of disease transmission. On panels E and F, large blue dots indicate strains that are transmitted following a stochastic transmission event (see the Model section). Given their longer duration, chronic infections lead to more transmission events than acute infections. More importantly, viruses transmitted from chronic infections tend to have higher replication rates due to within-host evolution (Figures 1E, 1F and S2), which could affect the epidemiological dynamics. In our approach, this effect will be averaged because we do not model the timing of transmission explicitly.

**Figure 2:**
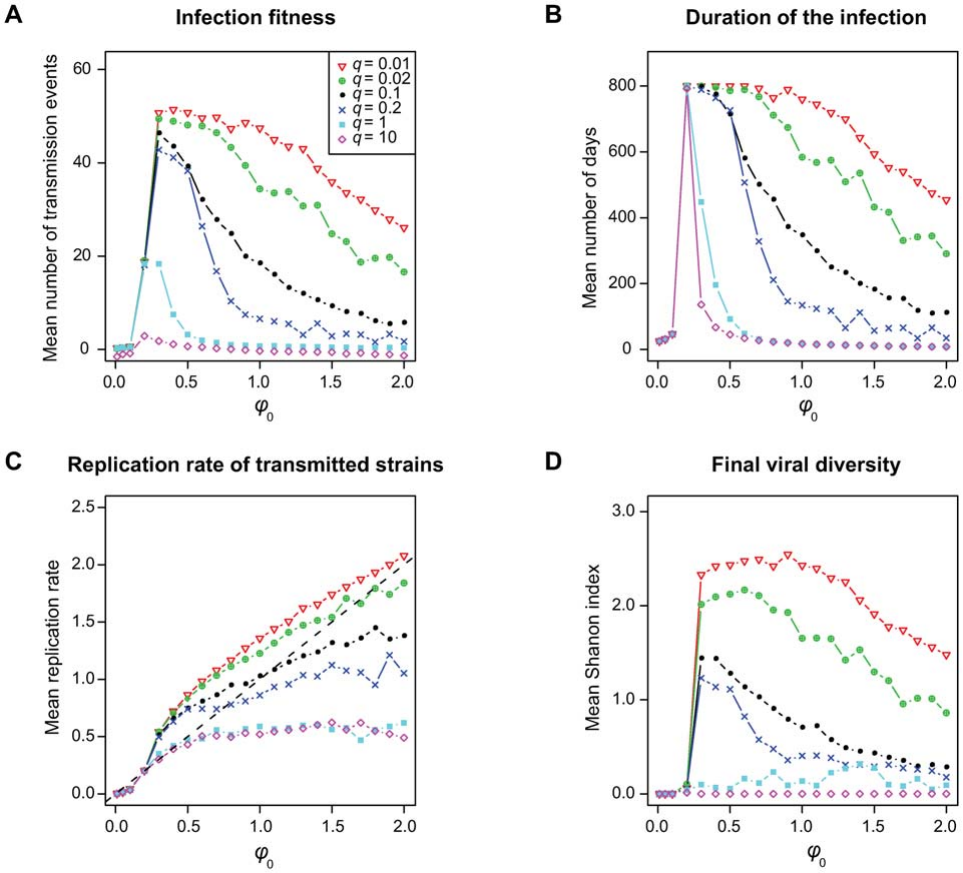
Effect of the variation of the initial replication rate (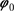) and of the breadth of cross-reactive immunity (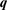) on infection life-history traits. A) Number of transmission events (i.e. infection fitness), B) Duration of the infection, C) Average replication rate of transmitted strains, and D) Viral diversity (Shannon index) near the end of the infection. Curves with different colour symbols represent simulations performed with different values of 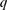 (see the box in panel A). The case with default parameter values is shown with black filled circles. For further details about the simulations see the main text and Text S1.

To get a more accurate picture of the evolutionary dynamics, we performed multiple runs of simulations with different parameter values. Table 2 summarises the effects we observe on infection life-history traits when varying parameters or initial values. Results are based on a linear fit using a multivariate linear model on the data we obtain (see Text S1 for further details on the simulations and on the statistical tests). Figure 2 shows the effect on infection life-history traits of variations in the initial replication rate (on the horizontal axis) and for different intensities of cross-reactive immunity (the different curves). The case with default parameter values (used for Figure 1) is shown with black filled circles.

**Table 2:** Influence of model parameters and of the initial replication rate on infection life-history traits.

Parameters ----------- Traits	Initial replication rate ( 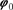 )	Cross-reactive immunity ( 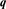 )	Maximum immune activation rate ( 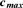 )	Mutation rate ( 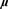 )	Mutation width of 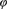 ( 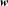 )	Maximum killing rate ( 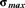 )	Treatment intensity ( 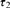 )
Infection fitness	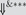	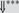	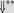	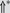	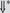	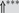	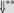
	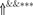						
Duration of the infection (Log)	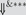	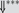	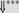	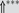	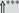	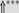	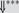
	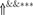						
Mean replication rate of transmitted strains	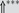	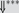	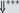	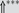	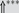	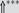	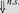
Final viral diversity	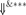	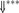	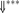	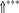	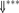	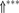	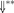
	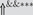						
Final number of immune cells (Log)	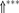	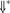	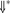	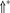	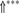	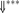	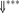
Final number of infected cells (Log)	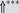	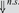	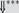	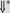	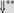	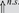	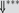

We performed linear fits based on a multivariate linear model on the mean values of the traits as a function of 12 factors: 9 model parameters (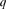, 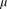, 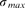, 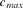, 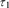, 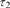, 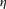, 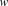, 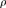), the initial replication rate 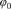, the initial number of precursor of lymphocytes (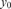), and the initial number of infected cells (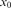). Further details about the statistical tests are available in Text S1. Significance level 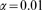.

Significance test codes: ‘^***^’: 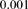; ‘^**^’: 0.01; ‘^*^’: 0.05; ‘*^n.s.^*’: non significant.

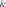
Test performed conditioning on 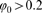.

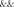
Test performed conditioning on 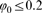.

### Role of the initial replication rate

As described in the Model section, in order to show that natural selection can act on the replication rate of the infecting strain at the epidemiological level, we need to show that this trait affects infection fitness and is heritable from one infection to the next. In this subsection, we focus on the default case (in black in Figure 2).

We find that the initial replication rate (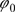) has a strong effect on infection fitness, which is maximised for low replication rates (Figure 2A). The fact that very low or high values of 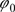 lead to low fitness values is likely to be due to short duration of the infections (Figure 2B). This is also clear from the statistically significant correlations we observe between the infection fitness and the initial replication rate (Table 2). Note that the value of 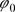 maximising the infection fitness depends on the base-line clearance term of infected cells (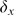, figure not shown). The correlation between 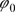 and the average replication rate of transmitted strains (Figure 2C) suggests that the trait is heritable. For low values of 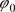, transmitted values can be higher than 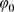 (Figure 2C) because the trait evolves for a longer time within the host (Figure 2B). Also, viral diversity increases as the infection progresses (Figure 1C), implying that the distribution of transmitted replication rates also changes over time (see Figure S1). According to these results, predictions are more complex to achieve for long-lasting infections and call for an explicit epidemiological model based on the distribution of transmitted strains resulting from the within-host dynamics.

Figure 2D shows that viral diversity near the end of the infection is maximised for low replication rates, which is likely to be due to an increased duration of the infection (Figure 2B). The density of infected cells near the end of the infection also has a maximum value for low replication rates (figure not shown). Finally, higher initial replication rates always lead to higher final levels of immune cells densities (Table 2). These results suggest that for low replication rates, infections are long lasting (most of the simulations end because the maximum time of 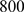 days is reached) whereas for other values the infection is often cleared by the immune response. Overall, the initial replication rate significantly influences the course of the infection.

### Role of cross-reactive immunity

We model the variation in the breadth of the immune response by varying the width (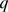) of the cross-reactive immunity function in equations 2a and 2b (see the Model section). Increasing the width (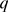) reduces infection fitness (Figure 2A and Table 2) and decreases viral diversity near the end of the infection (Figure 2D). The decrease in fitness seems to be due to a decrease in the duration of the infection (Figure 2B). Increasing cross-reactive immunity (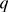) also decreases the replication rate of transmitted strains (Figure 2C). This result, which may seem counter-intuitive, is a consequence of the stochastic nature of our simulations. Increasing 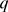 increases the rate of immune activation, which limits the opportunities for escape mutations and thus decreases the duration of the infection (Figure 2B). In our model, however, the activation rate of immune cells depends on the replication rate of the virus. Therefore, an increase in immune pressure confers a strong selective advantage to slow-replicating strains.

In the case of infections caused by a rapidly replicating strain (high 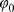), there are two scenarios: rapid clearance or rapid evolution towards lower replication rates. The latter prolongs the infection and the number of transmission events, thus decreasing the average value of replication rates of transmitted strains. Unless cross-reactive immunity is very high, diversity near the end of the infection is maximised for low values of the initial replication rates (Figure 2D). Overall, cross-reactive immunity significantly decreases infection fitness and within-host evolution, and alters the strain composition and therefore the transmission dynamics.

### Role of the mutation process

**Figure 3:**
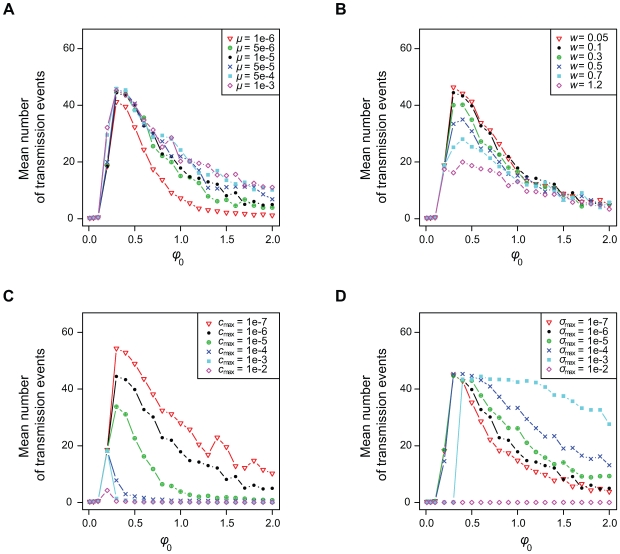
Effects of parameter variations on infection fitness. Effect of the initial replication rate (x-axis) on the infection fitness for different values of A) the mutation rate 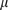, B) the mutation width for the replication rates 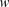, C) the maximum proliferation rate of immune cells 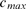, and D) the maximum killing rate of immune cells 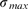. Different parameter values are shown using different colour symbols (see the box in each panel) and the default case is in black. For further details about the simulations see the main text and Text S1.

We model viral mutation as a stochastic process (see the Model section and Text S1). A newly generated strain is identified by at least one new trait value (its replication rate and/or its antigenic value). We study the effect of the mutation process on infection life-history traits. Overall, there is no substantial effect of the mutation rate (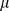) on viral fitness, which has a maximum value for low values of the initial replication rate (Figure 3A). However, increasing 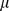 tends to be slightly deleterious in terms of infection fitness for strains with low 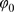 because, with high 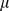, rapidly replicating mutants tend to appear earlier, which decreases the duration of the infection. For high values of 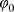, however, increasing the viral mutation rate leads to an increase in viral fitness because mutation rate increases the probability that an escape mutant is generated before the first infecting strain is cleared. The mutation rate also significantly correlates with the duration of the infection, the final level of viral diversity and the average replication rate of transmitted strains (see Table 2 and Supporting Figure S2).

Increasing the width (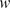) of the uniform distribution from which new mutant replication rates are chosen decreases infection fitness and increases both the duration of the infection and the initial replication rate that maximises fitness (Figure 3B and Table 2). Also, increasing 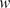 leads to a significant increase in average replication rate of transmitted strains (Figure S2) and to a decrease in final viral diversity (Table 2). Contrary to the mutation rate, increasing the width 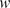 does not increase the probability that an escape mutation is generated by the initial strain. However, for high values of 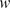, rapidly-replicating mutants appear early in the infection. Since these mutants are more adapted to the apparent competition taking place at the within-host level (see the Discussion), increasing 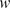 decreases the infection diversity and fitness for low values of 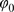. From a biological point of view, increasing the size of the mutation step can be interpreted as the introduction of recombination between unrelated strains. This process is rare for HCV, but it has been shown to occur within some hosts [49].

### Role of immune system activation and killing rates

Increasing the maximum T-cell activation rate (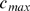) improves the immune response, therefore the consequences of an increase in 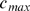 are not surprising: it significantly decreases infection fitness (Figure 3C), duration of the infection, final diversity and final total viral load (Table 2). Also, the initial replication rate that maximises infection fitness increases with 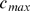, which is consistent with earlier models without within-host evolution [5]. For large values of 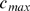 infections tend to be very short and there is no heritability of the trait (see Figure S2).

Increasing the maximum T-cell killing rate (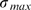) also increases the efficiency of the immune response but the evolutionary response seems to differ with respect to variations of 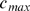 (Figure 3C and D). Increasing 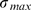 significantly increases both the fitness of rapidly replicating strains and the duration of the infections these strains cause (Table 2). Indeed, the increase in 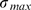 leads to a decrease in the number of infected cells early in the infection. Since immune activation is proportional to the number of infected cells present in the system, the increase in killing capacity also has a drawback: it slows down the immune response. Therefore, both the duration of the infection and the viral density increase, thus improving the infection fitness. When the killing rate is very high, the virus is completely eliminated and no within-host evolution occurs. Overall, these results show that interfering with immune activation or immune killing can lead to different outcomes. Also, the result that increasing immune response does not always reduce viral fitness is in accordance to the experimental observation that HCV generates a highly cytotoxic environment and therefore it is the immune response that may jeopardise host survival. Our model thus provides a simple mechanism that explains how an increase in immune resources can limit the immune response.

We also studied the effect of the initial population sizes of infected cells and of lymphocytes (precursor frequency), the possibility of host death (i.e., virulence) and of saturation in the lymphocyte proliferation function in equation 1. These results are shown in Text S2.

### Long-term optimal replication rate

**Figure 4:**
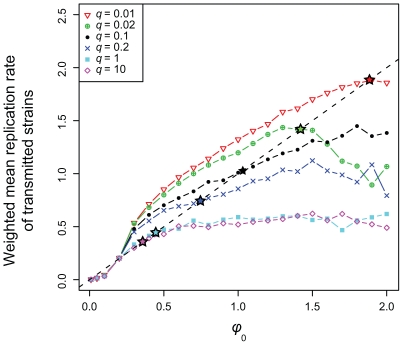
Weighted average replication rate of transmitted strains for different values of cross-reactive immunity (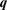). This figure is obtained by combining Figures 2A and 2C (see the main text). The average replication rates are weighted with the infection fitness values (see Figure 2A). Long-term optimal replication rates are indicated with stars, which are located at the intersection between the curves and the 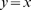 line. Intuitively, if the transmitted replication rate is above the 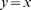 line, the virus evolves towards higher replication rates, whereas if the transmitted replication rate is below the line, the virus evolves towards lower replication rates. Only if the transmitted replication rate is on the line do we have an evolutionarily singular strategy. Increasing cross-reactive immunity decreases the long-term optimal replication rate.

In the absence of within-host evolution, Figure 2A would be sufficient to infer the evolutionary stable strategy (ESS, [50]) of the virus, i.e., the replication rate that is selected on the long term. In a model with within-host evolution where all infections would produce the same number of secondary infections (i.e., have the same infection fitness), Figure 2C could be used to find the ESS. The latter, if it exists, is at the intersection between the curve and the 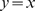 line (see the caption of Figure 4 for further details). Depending on the topology near the intersection, this singularity can be evolutionary stable (i.e., be an ESS) or unstable: if the curve intersects the 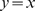 line from top to bottom (i.e. that it is concave), there is an ESS.

In our model, each one of the transmitted strains potentially has a different infection fitness based on its replication rate. We therefore combine differential fitness (Figure 2A) and within-host evolution (Figure 2C) to identify the ESS (see Model Section). Figure 4 shows the average replication rate of transmitted strains *weighted* by their infection fitness for different values of 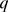. In the default case, the ESS is obtained for 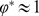 (the black star in Figure 4). This estimate is close to what would be obtained by ignoring the differences in infection fitness, which indicates that within-host evolution acts more strongly than between-host evolution. Increasing the level of cross-reactive immunity favours those strains with higher replication rates, which eventually dominate the population on the long term. For low values of 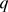, curves in Figure 4 strongly differ from those in Figure 2C. This shows that the control over trait evolution can shift from the within-host to the between-host level when parameter values change.

### Effect of treatments on viral evolution

We simulate two types of treatments: the first type improves the killing of infected cells and is modelled with an additional killing term of infected cells, the second type blocks viral replication and is modelled with a limitation term on viral replication. Current treatments of HCV use 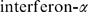 typically in combination with ribavirin. These treatments act on viral dynamics by blocking viral replication [51], but also stimulate innate and cellular immunity [52],[53].

**Figure 5:**
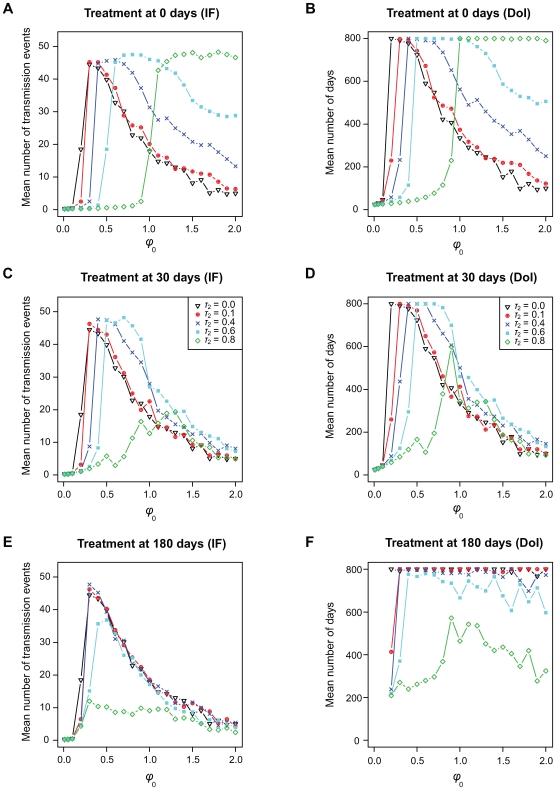
Effect of the timing and of the intensity of anti-viral treatments limiting viral replication. In panels A, and B the treatment starts at the time of the infection, in panels C, and D the treatment starts 30 days after the beginning of the infection. Finally in panels E and F treatment starts 180 days after the infection. Panels A, C, and E show the average infection fitness (IF). Panels B, D, and F show the average duration of the infections (DoI). The latter is calculated by excluding simulation runs that result in host before the beginning of the treatment (this is done to highlight the effect of the treatment at the within-host level). Different colours correspond to different treatment intensities (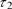, see legend in panels C and D). The default case is in black. Note that in panel F, infections starting with low replication rates are cleared before treatment is initiated. For further details about the simulations see the main text and the Text S1.

In Figure 5, we show the effect of a replication-blocking treatment administered with different intensities (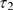) and started at different time points (0, 30 or 180 days after infection). A treatment model based on increasing immune killing leads to similar results (results not shown). This is because, in our model, there is only immune-mediated competition between strains and no resource competition. Overall, increasing 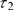 decreases the infection fitness, the duration and the diversity of the infection (Figure 5A, C and, E, and Table 2). Note that the average infection fitness values we show in Figure 5 are obtained by including hosts who cleared the infection before the initiation of the treatment. Regardless of the starting time, increasing the treatment intensity increases the value of the initial replication rate that maximises infection fitness (Figure 5A, C and E), as in models without within-host evolution [5].

The timing of the treatment also affects the course of an infection. When the treatment is initiated at the onset of an infection (which would be the case if hosts are treated following exposure), we see a shift of the curves to the right (Figure 5A, C and E). This means that the virus can compensate completely for the fitness decrease due to treatment by increasing its replication rate. The current practice in HCV infections is to treat the patient after several months to avoid treating cases that resolve naturally, and because treatments based on 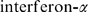 are toxic. We show that delaying the treatment also has an evolutionary advantage because strains that do well in a treated host are also the strains that tend to lead to acute infection (Figure 2B).

In order to investigate the effect of treatment at the host level rather than at the host population level, we focussed on the duration of the infection and removed from the analysis hosts who had cleared the infection before the beginning of the treatment (Fig. 5B, D and F). We find similar effects as for the infection fitness: depending on the initial replication rate and on the treatment intensity, the duration of the infection can decrease (e.g., if 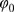 is low and 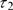 is high) but it can also increase (e.g. if 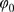 is high). The only case where the treatment always decreases the duration of the infection is when treatment begins after 180 days (Figure 5F). This is because in the other cases not all the acute infections have ended and treating at that time allows rapidly-replicating strains to persist in the host.

## Discussion

We developed a nested model that incorporates within-host evolutionary dynamics into an epidemiological perspective to study the evolution of viral traits, such as the replication rate. This approach raises an important issue in that viral traits evolve within the host during the course of an infection and at the between-host level. In other words, what infects a host (a strain with its initial replication rate) differs from what is transmitted (a distribution of replication rates). We show that the replication rate of the strain that initiates the infection has a strong effect on the course and on the fitness of an infection. On average, the initial replication rate tends to be heritable from one infection to the next. These two results (heritability and fitness effect) combined with the known variability in replication rates indicate that, in our model, natural selection acts on viral replication at the between-host level.

### Slow replication maximises infection fitness

We find that infection fitness (measured as the number of transmission events per infection) is maximised for infections initiated by slow-replicating strains. This is partly due to the assumption we make on the immune proliferation function, which allows slow-replicating strains to have the most efficient resource exploitation (by escaping from the immune response). Escaping from the immune response has a second advantage because it gives more opportunities to these strains to generate escape mutants. We are not aware of studies on the evolution of HCV replication rate at the between-host level. In the case of HIV, the subtype C of HIV-1 has been shown to be spreading more rapidly than other subtypes [17]. Interestingly, viruses of this subtype are out-competed when grown with viruses from other subtypes. Even if *in vitro* conditions have little in common with the within-host environmental conditions, these results suggest that slow replication could be optimal at a population level for rapidly evolving viruses (see also [54]).

In epidemiology, the idea that low replication rates can be optimal is not new; it is present in the transmission-virulence trade-off theory [55],[56], which states that pathogens should spare their host to maximise the duration of the infection. However, most models ignore within-host evolution of the replication rate (but see [25]). Our nested model provides an explicit description of the within-host mechanisms that drive evolution toward low replication rates.

Our model also illustrates the conflict between levels of selection [25]–[27]. At the between-host level, slow-replicating strains have an advantage because they tend to generate longer infections, thus leading to more transmission events. At the within-host level however, rapid replication is favoured through cross-reactive immunity. As discussed in [8], rapid replication leads to higher activation of the immune response, which penalises slow-replicators (rapid replication rate being a means to compensate for the immune killing). Note that here, contrary to other models, the virus cannot indefinitely evolve to higher replication rates within the host because the immune activation function depends on both viral density and viral replication rate [7]. This explains why, for default parameter values, strains with a replication rate close to 1 are likely to dominate the population over the long term even though the highest infection fitness is reached for replication rates close to 0.3. Moreover, increasing cross-reactive immunity favours more slowly replicating strains in the long term at the between-host level. This effect is much clearer at the between-host level (Figure 4) than at the within-host level (Figure 2A), and also highlights the fact that changes in within-host parameter values can shift the overall selective pressure from the within-host to the between-host level.

### Importance of the timing of treatments

Our model allows us to study the evolutionary consequences of anti-viral treatments on infection life-history traits. Increasing the efficiency with which a treatment blocks viral replication decreases viral fitness, but also increases the evolutionarily optimal replication rate of the virus. This is consistent with theoretical [57],[58] and experimental results [59]: treatments decrease viral fitness but they also select for more virulent parasites.

Most epidemiological models find a host ‘selfish’ strategy, which consists in increasing its own protection at the expenses of the community [57]. Here, starting a treatment is not always the best option for the host because, in addition to the toxicity of the drugs, treatment can increase the duration of the infection. This is especially true for acute HCV infections, where hosts recover without intervention. Therefore, we stress the importance of a neglected factor in evolutionary epidemiology, i.e., the timing of treatment administration. We show that administering treatment a few months after the infection begins, imposes a treatment-free period that selects against rapidly replicating strains. Of course, this procedure is acceptable only if the short duration of the infection comes from rapid host clearance and not from host death (fortunately, acute HCV infection very rarely results in fulminant disease). These predictions could be tested empirically by comparing the course of the infection in patients infected by similar HCV genotypes for whom treatment started at different time points.

### Viral evolution and disease outcome

Few studies have attempted to model HCV disease outcomes. Wodarz [60] studied the pathology of HCV with a multi-strain model that described both cellular and humoral immune responses. However, this model did not account for antigenic diversity nor for different viral traits. Population genetics and phylogenetic analysis applied to HCV provide evidence that HCV evolution within-host is under a strong immune pressure [61] and that disease outcome is statistically associated with the number of genetic sites selected [62].

A key feature of our model is that for the exact same parameter values, we can observe chronic or acute infections (for a review on this topic, see [63]). In both cases there is a significant amount of viral diversity generated, which means that this result is not only due to the fact that fewer escape mutants are generated during acute infection outcomes. Finally, we find that there is a delay of a few weeks in launching a specific immune response following viral infection, which is consistent to reports in acute HCV infections. This also echoes a more general concern about most within-host models, in which the outcome of the infection tends to be an assumption rather than a result [38].

We show that replication rate of the initial strain and the parameters describing the immune response alter the probability that one of the two outcomes (chronic or acute infection) is reached. The few observations on the correlations between the outcome of HCV infections and the viral replication rate tend to support our results. Data on the growth of HCV in sera suggest that slow replicating viral populations are common in HCV cases with viral persistence [64]–[66]. A direct relationship between the initial viral replication kinetics and cellular responses has also been observed [46]. Moreover, a study based on a peculiar sample of seven people infected on the same day from the same source showed a correlation between viral load and the dominance of a few strains [67]. They also showed that subjects with lower viral loads had a higher diversity that increased over time, thus suggesting an evolutionary process driven by slow-replicating strains, which fits with our result that these strains have the highest infection fitness.

The immune response against HCV is puzzling. The mechanisms responsible for the high rate of viral persistence are thought to be the result of complex early host-virus interactions that involve immune system heterogeneity, viral diversity and cross-reactive immunity [63],[68]. In acute HCV infection there is a significantly broader cytotoxic T-cell response with wider variant cross-recognition capacity than in chronic cases [69]. A significant result of our work is that cross-reactive immunity decreases viral diversity, and infection fitness, thus supporting the idea that viruses face a trade-off: low replication maximises infection fitness but rapid replication helps to escape from cross-reactive immunity. However, one needs to be careful about drawing conclusions from our model regarding the optimal level of cross-reactivity of the immune response, as increasing the width of the cross-reactive immunity function confers a cost-free advantage to the immune system. Therefore, it should not come as a surprise that infection fitness decreases with cross-reactive immunity. A more detailed analysis should introduce a cost to the breadth of the immune response (for instance in terms of efficiency of killing).

### Perspectives

The between-host dynamics of our model could be extended in several ways. We used an invasion fitness analysis (with 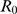), which simplifies the analysis but can also be misleading [70]. Adding a detailed between-host framework would allow to remove the assumption that the size of the susceptible host population remains constant and would lead to more accurate epidemiological dynamics.

Modelling the between-host dynamics more explicitly would allow one to take into account the age of the infection. Day [71] shows that variations in transmission rates during the infection (which is likely to happen if parasite densities vary) can affect the epidemiology of the disease. Here, as we show in Figures 1E and S2, the trait of the transmitted strains varies over the duration of the infection. This could significantly impact on the evolution of the trait during an epidemic, for instance if rapidly replicating strains are transmitted later in the infection (see [6] for a similar discussion in a case with only two strains per host).

Another extension would be to include several transmission routes for the virus. The model we use here assumes that infections are initiated by a unique viral strain, which is known to be the case for sexual transmission of HIV [72]. However, transmission can also occur through needle sharing in which case the inoculum is more likely to be diverse. Allowing for multiple transmission routes would influence both the within- and between-host dynamics.

Regarding the selective forces involved in the evolution of HCV at a population level, there is evidence that the major histocompatibility complex (MHC) allele diversity among the population is a major force driving the evolution of the virus [68]. Host heterogeneity could be introduced by assuming that each host type has lymphocytes with different antigenic values for their receptors. This would allow us to identify the optimal viral strategy at the between-host levels for different parameter sets. It would also allow us to further investigate the unresolved issue of why, during HCV infection, mutants that escape the immune responses do not always revert to wild type forms upon transmission. Here, we suggest that this evolutionary process may be influenced by both the antigenic value (epitope escape) and the viral replication rate; host diversity is likely to have a strong impact as well.

Our work leads to predictions that can be tested experimentally: strains that dominate early in the infection and those that dominate later have different replication rates; and the replication rate of the initial strain and the mutation rate affect the duration and/or the fitness of an infection (see Table 2 for a summary). Of course, some experimental difficulties need to be overcome. First, estimating the replication rate of a circulating strain may be challenging. Second, diversity in replication rates may be difficult to assess if some of the circulating strains are rare. Third, in the case of HCV, it is rare to detect the infection in the early acute phase (as it is typically asymptomatic). Another interesting question raised by this model that calls for empirical support is the heritability of infection life-history traits in the case of rapidly evolving diseases. This question has been studied in the case of HIV virulence, but there is still no convincing evidence that this trait is heritable from one infection to the next [54].

This study stresses the key role played by the cross-reactive immune response in controlling the duration and the fitness of an infection. In addition to the theoretical challenge described above, an experimental validation of this study could be to measure *ex vivo* the strain-specific immune response and the degree of cross-reactive immunity within-host, and finally to investigate the role of these responses in the outcome of the infection. This model validation could be achieved by identifying strain specific and shared T-cell responses across multiple strains during an infection. Experimental assays such as the Elispot assay, *in vitro* stimulation and intracellular cytokine detection, and MHC-tetramer staining are established methods to measure the T-cell responses against specific T-cell epitopes [69],[73],[74]. However, these assays carry a substantial cost given the large uncertainty and variation in the distribution of MHC class I and II epitopes between infected individuals. Bioinformatics tools have been successfully used to predict pools of MHC class I epitopes (which can be synthesised as peptides) to be employed in immunological assays to measure T-cell specific responses [75]; these predictions minimise the experimental effort in identifying T-cell epitopes in terms of cost and timing [76]. One could validate the model predictions by measuring variations in viral replication rates with *in vitro* assays, and of strain specific immune responses using prediction tools and T-cell response read-out techniques, during the course of the infection. Overall, combining bioinformatics tools, immunological assays, and theoretical models on viral trait evolution forms a promising framework for detecting and measuring the presence of immune selection during the within-host evolution of rapidly mutating viruses.

## Model

### Within-host dynamics

We model viral dynamics with an immune control model based on a predator-prey-like interaction, where lymphocytes are predators. We assume a finite number of T-cell clones per host, 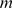, while the number of viral strains, 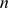, varies during the infection. We explicitly take into account the cross-reactivity of the immune response i.e., the fact that a lymphocyte clone can be activated by and destroy more than one viral strain.

Following previous models, we simplify the virus life cycle by focusing on infected cells only [37],[38]. The reason for this is that we study a case where viral growth is limited by the immune response, not by resource availability (indeed, only a fraction of the total amount of liver cells seem to be infected, [77]). Moreover, the half-life of free viruses has been shown to be low [51], which supports the assumption that these dynamics can be considered to be at equilibrium. Mathematically, if we denote the number of cells infected by viral strain 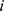 by 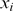 and the number of T-cells of type 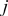 by 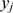, the population dynamics are governed by the following system of 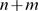 equations: 
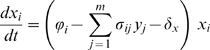
(1a)

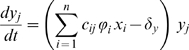
(1b)where 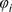 is what we refer to as the replication rate of strain 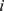 (it corresponds to the rate at which viruses of strain i are produced and infect susceptible cells), 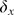 is the base-line death rate of infected cells, 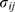 is the killing rate of cells infected by viruses of strain 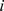 by lymphocytes of clone 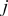, 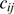 is the activation rate of lymphocytes of clone 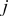 by cells infected by viral strain 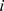 and 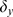 is the lymphocyte death rate (the notations used are summarised in Table 1). As in Alizon [7], the immune activation term does not only depend on the number of infected cells (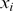) but on the overall viral growth rate (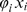, see Introduction).

T-cells of clone 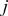 are defined by one trait that does not vary over time: their receptor (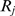). Viruses of strain 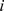 are defined by an antigen value (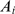). In reality, viruses are recognised by cellular immune responses via multiple antigenic peptides but the combination of peptides is unique and it is this combination that our antigen value reflects. For simplicity and to avoid boundary effects, the receptor and antigen values are chosen on a finite space, i.e. in 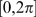. The intensity of the cross-reactivity between a lymphocyte clone and a viral strain depends on a measure of the genetic proximity, i.e. 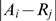. The strength of the cross-immunity between an antigen 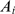 and a receptor 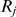 is then given by a 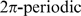 function. More precisely, we have
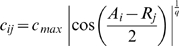
(2a)

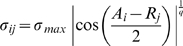
(2b)where 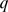 is the width (or breadth) of the cross-reactive immunity and 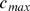 and 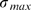 are the maximum values for 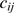 and 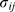, respectively. We assume that cross-reactivity affects both the T-cell proliferation and the killing of infected cells. Here, increasing 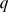 results in an increase in the range of antigen values 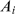 recognised by receptor 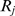. Note that the cross-immunity function has a maximum for 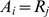. Note also that in the model as we define it in equation system 1, a viral strain undergoes immune killing by all the lymphocyte clones (but with variable efficiency), which means there is never complete immune escape. For further details on modelling cross-immunity as a function of the distance between antigens and receptors, see [8] and the discussion therein.

### Evolutionary dynamics

Equation system 1 only describes within-host dynamics, but nothing is specified concerning evolutionary processes. Here, the number of viral strains 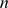 varies due to stochastic mutations. Newly infected cells can mutate to a new viral strain with probability 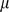. Mutations are modelled as stochastic events following a Binomial distribution (for details see Text S1).

In this model, viruses of strain 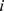 are defined by two traits, their replication rate (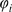) and their antigen (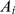). Each time a virus replicates within a cell, it produces ‘mutants’, i.e. offspring with different genomes. Mutations in the genome sequence can be silent or affect the phenotype of the virus, and then can be deleterious or advantageous. Here, we consider mutations affecting both the antigen and the replication rate of a virus. Therefore, our mutation event is a composite event that accounts for mutations leading to a new replication rate and to a change in the antigenic value.

We analyse the effect of escape mutants with a hybrid stochastic-deterministic approach (see Text S1), where the population dynamics of the strains are given by equation system 1. A mutant strain is defined, as any other strain, by its antigen value and its replication rate. The antigen is drawn randomly among one of the 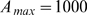 possible values (see above). By doing so, we allow for the mutant to (partially) evade the existing immune response while limiting the impact of the antigen value on viral evolution. The mutant replication rate depends on that of the original strain. There is little data on the shape of the distribution in which the trait value of a new mutant should be drawn, even if there are strong constraints on RNA genomes [78]. Without further information, we assume for simplicity that the replication rate of the new strain is drawn from a uniform distribution of width 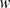 centred at the replication rate value of the original strain. We assume a finite number of replication rates 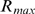 that is fixed to 100 in the default case.

We wish to stress that our model allows for backwards mutation. By this we mean that a mutant can be identical to another strain in the infection or to a strain that was present earlier in the infection. This approach provides a realistic representation of the quasi-species characteristics of HCV evolution and frees us from potential biases due to infinite allele model assumptions.

The number of strains is not constant in these simulations. We follow the evolution of strain diversity by measuring the Shannon index, 
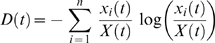
(3)where X(t) is the total density of infected cells at time t, i.e. 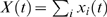. The higher the value of 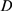, the more diverse the infection is.

We extended the model to study the effect of two types of treatments. The first type of treatment directly increases the killing of infected cells. It is obtained by adding a death term (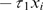) in equation 1a, where 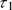 is the treatment efficiency rate. The second type of treatment blocks viral replication. This is modelled by multiplying the 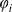 by 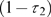 in equations 1a and b, where 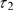 is the treatment efficiency in reducing viral replication. Treatment efficiencies are assumed not to vary among strains. Further details concerning the model with treatments can be found in the Text S1.

We do not introduce host death in the default case of the model. The main justification for this assumption is that, from the point of view of the virus, host recovery or host death are very similar, the only difference lies at the epidemiological level that is outside the scope of this study. The effect of virulence is shown in Text S2. We also investigated the effects of a saturation term in the lymphocyte proliferation rate due to resource competition. This term does not change qualitatively the results presented (see Text S2).

### Evolutionary epidemiology

Having a nested model requires a careful definition of the viral trait that evolves at the between-host level. Here, we follow the replication rate of the strain that causes an infection (or ‘initial replication rate’). Can this trait evolve under natural selection at the between-host level? For this, three conditions must be fulfilled [79]: the trait value must be variable in the population, it must be heritable (here from one infection to the next) and it must have a fitness effect. The first condition is fulfilled because viral replication rates are known to vary among infections (see the Background section). The validity of the last two conditions is unknown: so far, no data show that the initial replication rate is heritable from one infection to the next, nor that it has an effect on the infection fitness. Our model allows us to test if these two conditions are fulfilled. If so, we can identify trait values that are optimal at the between-host level.

Now that we have specified our trait of interest, we need to introduce a fitness measure for the infection bearing the trait. The ‘fitness’ of an infection can be expressed through the basic reproduction ratio (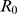), which indicates the number of new infections caused by an infected host in a population of susceptible hosts [9]. This 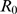 is a measure of the invasion fitness and, as discussed in [70], it can be used to study disease evolution provided that the epidemiological dynamics are simple (for instance there should not be frequency-dependent feedbacks).

To estimate the infection fitness, we introduce a random transmission event in the simulation. At each time step, this event occurs with a probability proportional to the log of the total density of infected cells at this time, thus reflecting recent data showing that the transmission rate during primary infections with HIV increases linearly or more than linearly with the viral load [80] (for further details, see Text S1). The trait of the transmitted strain depends on the proportion of each type of strain at the time the transmission event takes place. We use the number of transmission events during an infection to estimate infection fitness. (This number correlates with the total number of viruses produced during an infection.) We also have access to the average replication rate of transmitted strains as a function of the initial replication rate, which allows us to assess the heritability, from one infection to the next, of the trait of interest.

In addition to the infection fitness, we investigate the effect of parameter values and initial values on several life-history traits of the infection: the duration of the infection, the final viral diversity (measured near the end of the infection), the final total immune cell density, and the final total viral load. The analysis is performed by varying one of the parameters at the time and running simulations for 22 different initial replication rates (ranging from 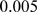 to 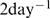). In order to keep the computational time within feasible time scales, we introduce a maximum duration of an infection of 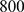 days (2 years circa), which is sufficiently long to represent a chronic infection. An additional reason not to prolong *in silico* infections beyond 800 days is that long-lasting infections are characterised by impaired immune responses, where the HCV directed immune responses undergo a functional change [21], which would call for a different modelling approach. To test the robustness of our results, we run each simulation setting 200 times (see also Text S1 for more details of the simulations).

Finally, we introduce a *weighted* average replication rate of transmitted strains. The rationale for this is that the average replication rate of transmitted strains *per se* is not sufficient to estimate the long-term fitness. For instance, in a very extreme case where only two strains (A and B) would be transmitted from an infected host and where the infection fitness of strain A would be close to 0, it is easy to see that in the long term strain B should be more frequent than strain A. In other words, if one of the transmitted strains generates an infection with negligible infection fitness, it will not contribute to shaping the virus population in the long term. If the infection fitness of an infection caused by a strain with replication rate 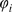 is denoted 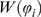, the weighted average replication rate is given by 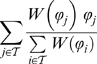
(4)where 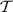 is the set containing all the strains transmitted from the host. Note that in the classical average, the weights 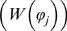 are assumed to be equal to 1. Similar methods, such as weighting the contribution of the transmitted viruses with their relative value of fitness at the next generation, are often adopted in evolutionary biology in kin selection models, where the fitness gain obtained by an individual through its offspring (in terms of inclusive fitness) has to be weighted by the reproductive value of the offspring [81].

## Supporting Information

Figure S1Dynamics of the average replication rate of transmitted strains and of the total number of strains transmitted per day We show the results obtained for 200 simulation runs for low, average, and high cross-reactive immune responses, respectively (*q*, indicated on the top of each panel). Other parameter values are as in Table 1, and 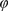 = 0.3. The top panels show the replication rates transmitted over time (red dots), and the solid line represent the result of linear regression (A) *r* ^2^ = 0.902, B) *r* ^2^ = 0.883, C) *r* ^2^ = 0.810). Bottom panels show the number of transmission events over time. Increasing cross-reactive immunity (from left to right) decreases the average replication rate of transmitted strains.(0.16 MB TIF)

Figure S2 Effects of parameter variations on the replication rate of transmitted strains. Effect of the initial replication rate *ϕ*_0_ (x-axis) on the average transmitted replication rate for different values of A) mutation rate *μ*, B) mutation width *w* of the replication rate, C) maximum proliferation rate of immune cells *c*_max_, and D) maximum killing rate of immune cells *σ*_max_. Different parameter values are shown using different colour symbols (see the box in each panel) and the default case is in black.
(0.52 MB EPS)

Text S1 Details about the simulations. This text file gives further details about the simulations and the statistical tests used in Table 2.
(0.08 MB PDF)

Text S2 Additional results. We discuss the effect on life-history traits of variations of the initial value of immune cells and infected cells, of the probability of virulence and of the effect of a saturation term for the description of lymphocyte proliferation rate function.
(0.08 MB PDF)
