# Supplementary figures and images for: The Evolutionary Dynamics of a Rapidly Mutating Virus within and between Hosts: The Case of Hepatitis C Virus
Source: PLoS Comput Biol. 2009 Nov 13;5(11):e1000565. doi: 10.1371/journal.pcbi.1000565 (PMC2768904; doi:10.1371/journal.pcbi.1000565)

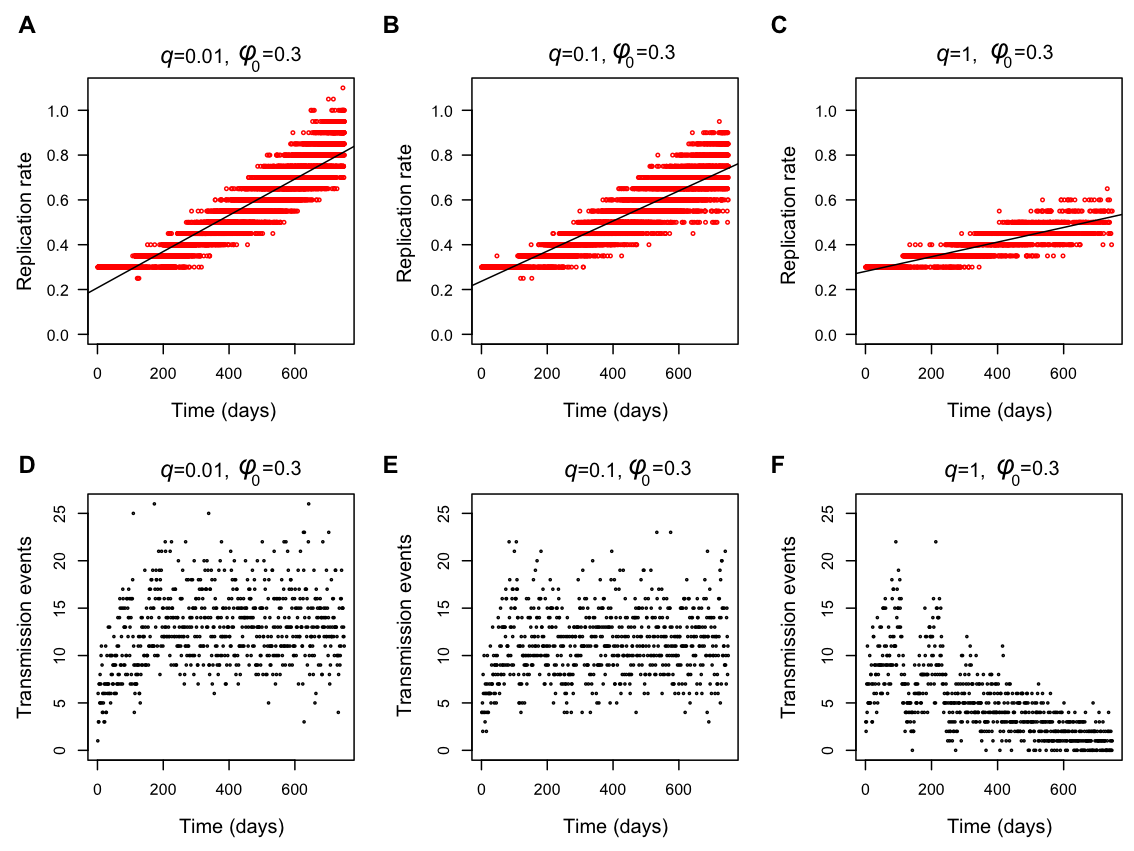

Supplement: Figure S1 — Dynamics of the average replication rate of transmitted strains and of the total number of strains transmitted per day We show the results obtained for 200 simulation runs for low, average, and high cross-reactive immune responses, respectively (q, indicated on the top of each panel). Other parameter values are as in Table 1, and = 0.3. The top panels show the replication rates transmitted over time (red dots), and the solid line represent the result of linear regression (A) r 2 = 0.902, B) r 2 = 0.883, C) r 2 = 0.810). Bottom panels show the number of transmission events over time. Increasing cross-reactive immunity (from left to right) decreases the average replication rate of transmitted strains. (0.16 MB TIF) [file pcbi.1000565.s001.tif]
