# Supplementary material for: The Evolutionary Dynamics of a Rapidly Mutating Virus within and between Hosts: The Case of Hepatitis C Virus
Source: PLoS Comput Biol. 2009 Nov 13;5(11):e1000565. doi: 10.1371/journal.pcbi.1000565 (PMC2768904; doi:10.1371/journal.pcbi.1000565)
Supplement: Text S1 — Details about the simulations. This text file gives further details about the simulations and the statistical tests used in Table 2. (0.08 MB PDF) [file pcbi.1000565.s003.pdf]

Text S1: *Details about the simulations*

In this Supporting Text, we provide further details about the simulations of the stochastic model and about the statistical tests performed to assess the effect of the parameter of the model on the life history traits.

A.1 The model

We describe the within-host evolutionary dynamics with a hybrid stochastic-deterministic model. Similar approaches have been proposed to model within-host evolutionary dynamics in HIV, showing that one can assume a deterministic behaviour for the population of infected and immune cells whilst keeping a stochastic description of the viral evolution [1, 2]. Here, contrary to previous models, we track the dynamics of two viral traits: the replication rate and the antigen characteristic of each viral strain present in the host (for a similar approach with only two strains per host, see [3]).

Due to the complexity of the model, numerical simulations are performed with a code written in C that is available upon request to the authors. In the model, at each time step, the population size of each viral strain and each lymphocyte clone are updated using the system of differential equation 1 given in the main text. This system is solved numerically with a fourth order Runge-Kutta algorithm [4]. A viral strain is removed from the system if the number of cells infected by this strain falls below 1.

Each viral strain is characterised by a replication rate and an antigenic value (see the Model section in the main text). The immune cells are described as a population of a finite number of T-cell clones m each with a receptor that recognises antigens within a range of values defined by the function given in Eq. 2 of the main text. In the simulations we assume $m = 20$. We assume that infections are generated by a single strain with an initial replication rate φ_0 and an antigen value A_0. This assumption is supported by recent data for HIV infections [5]; little is know about HCV transmission dynamics.

Simulations are ended when the maximum time ($T_{max} = 800$ days) is reached or when the total numbers of infected cells and immune cells both reach a threshold value of 10^{12} that we assume a large value that correspond to the death of the host.

A.2 Mutation process

At each time point after infection, virus mutates with probability μ per day. The probability that a new strain emerges is drawn from a binomial distribution (similar to [2]) $B(\Delta x_i, \mu)$, where Δx_i is the number of newly generated infected cells from strain i. We here use the approximation $\Delta x_i \approx \varphi x_i \Delta t$. For each new strain we draw randomly the new traits (the replication rate and the antigenic value). The new replication rate is drawn from a uniform distribution centred around the replication rate of the strain generating the mutation and with a range of values (width) w. We also assume a maximum value for the replication rate, $\varphi_{max} = 5$. The new replication value is drawn from a finite set of values R_{max}, which is assumed to be 100 in the default case. A new antigen is drawn from a finite set of values, A_{max} that are uniformly distributed. Because the cross-immunity function we use is 2π-periodic, we assume that antigenic values are uniformly distributed on a circle $[0, 2\pi]$, thus antigens are multiples of $2\pi/A_{max}$. We simulate the case

for $A_{max} = 1000$ (see also Supporting Text S4). In the model we assume a finite number of replication rates and a finite number of antigenic values. Therefore there is a probability that newly generated mutants are already present within the host. For each new mutant, if the newly generated strain has both replication rate and antigen value already in the system in a previously generated strain, then we add these mutant cells to the population infected with the same type of virus. Otherwise, we increment the number of types $m(t) = m(t - \Delta t) + 1$ and the number of differential equations of system 1 in the main text accordingly.

A.3 Transmission dynamics

To assess the heritability of viral traits from one infection to the next, we simulate transmission events during the infection period. To do so, we assume a simple epidemiological model, where virus spread across an homogenous population of susceptible individuals. This assumption is analogous to that done to define the classical R_0 [6]. We assume that the probability of encountering a new susceptible host is homogenous and we neglect heterogeneity in the host population. In this model, we approximate the probability of transmission as a stochastic event that depends on the logarithm of the total viral load within the source. Recent data on HIV show that the probability of transmission during primary infections is at least a linear function of the logarithm of the viral load [7]. In our model, the production of viral particles is at a steady state, therefore the number of viral particle is proportional to the number of infected cells, which is the variable that we follow in the simulations (see the main text).

A simple way to model the probability of transmission is to assume a non-homogenous continuous Poisson process with rate $\lambda(t) \approx \rho \log(X(t))\Delta t$, where $X(t)$ is the total number of infected cells at time t within the source, $\Delta t = 1$ is the time interval between events in the simulations of the model, and ρ is a constant transmission probability (ρ is a free parameter that can be varied to reflect the environmental condition or genetics of the population considered). At any time step we consider the probability that a transmission event occur, which is simply 1 - the probability that no event occurs: $1 - e^{\lambda}$. On average, the time at which a transmission event occurs is $\frac{1}{\lambda}$. The default case is a situation where transmission events occur on average every 50 days. Upon transmission, we assume that only a single strain is successfully transmitted into the new host. The transmitted strain is chosen proportionally to the number of infected cells present in the host at time t, namely with probability $\frac{x_i}{\sum_j x_j}$. This strong bottleneck assumption is supported by data on HIV suggesting that 80% of transmitted events involves single types [5]. During the simulations we store the values of the traits of the transmitted strain and the time of the events.

A.4 Virulence events

In the default case we assume that hosts do not die (unless unrealistic values of viral load or immune response are reached (we used a threshold of 10^{12}). As explained in the text, we make this assumption because the scope of this work is to study viral evolution; therefore host death and clearance have a similar effect. However, for completeness, we investigate the effect of virulence in a more detailed analysis, where host death is a stochastic event that occur with a probability that is proportional to the viral load.

As for the transmission dynamics, we describe the virulence event as a stochastic event with a non-homogenous Poisson process. The probability of virulence event occurring at time t is $\lambda_{vir}(t) \approx \eta(X(t))$, where $X(t)$ is the total number of infected cells in the host at time t. Here the probability that a virulence event occurs is drawn in a parametric distribution (Poisson distribution). Virulence occurs at most once in an infection, it might be more appropriate to model it using a non-parametric probability distribution. However, our approach has the advantage of being a simple and intuitive way of modelling this event, assuming that it depends on the viral load. At any time point during the infection, we compute the probability of virulence as 1 minus the probability that no virulence event occurs. If a virulence event occurs, then the host dies and the simulation is stopped. We record the traits values and the time of death. Results are shown in Table 3 of Supporting Text 4.

A.5 Statistical tests for the correlation analysis on viral life-history traits

To assess the effect of parameter variations on the viral traits and their heritability, we perform statistical tests on multiple simulation runs, where each parameter setting was run 200 times. This value is sufficient to explore the variation for each parameter setting. We also perform simulations where we repeat 500 or 1000 times the same set of parameter values observing little variation in the range of values of life history traits (not shown).

Table 2 in the main document summarises the effect of the variation of 12 factors on the infection life-history traits. The factors we vary are the initial replication rate of the infecting strain (φ_0), the intensity of cross-reactive immunity (q), the mutation rate (μ), the width of the distribution of the replication rates (w), the maximum immune activation rate (c_{max}), the maximum killing rate σ_{max}, transmission rate (ρ), the virulence (η) and the treatment intensities (τ_1 and τ_2). We also study the effect of the initial viral load and of the precursor frequency of immune cells. Simulations are performed by varying only one parameter per setting and keeping all the others at their default values (see Table 1 in the main text). We simulate the model using a range of 22 values (between 0.01 and 2) for the initial replication rate φ_0 and with a range of 6 to 10 values for the other factors.

The statistical association analysis is conducted with a multivariate general linear model employing the function “lm” in the R package (freely available at http://cran.r-project.org/). In the model, we assume that each infection life-history trait depends on a linear combination of the 12 factors (see above). We also test statistical associations with a univariate linear model, which assumes dependency of the trait on a single factor. Tests are performed using the same factors. Results are very similar to those obtained with a general linear model (not shown).

A.6 Modelling host treatment

We investigate the effect of the two types of treatments on the life-history traits by adding the treatment efficiency rate as a further parameter of the multivariate linear model. For the treatment tests we simulate again 200 runs for each parameter settings and the treatment efficiency rates τ_1 and τ_2.

Equation system 1 with treatment becomes:

$$\frac{dx_i}{dt} = \left(\varphi_i (1-\tau_2) - \tau_1 - \sum_{j=1}^{m} \sigma_{ij}\, y_j - \delta_x \right) x_i \tag{3a}$$

$$\frac{dy_j}{dt} = \left(\sum_{i=1}^{n} c_{ij}\, \varphi_i (1-\tau_2)\, x_i - \delta_y \right) y_j \tag{3b}$$

These equations illustrate the difference between τ_1, which mimics killing of infected cells, and τ_2, which blocks viral replication, which also affects the proliferation rate of the lymphocytes because it depends on φ_i. Table 2 shows the result of the test performed on τ_2. Similar results are obtained from simulations where we vary τ_1. For both treatment types we simulate several strategies of administration: the treatment can be initiated as early as the infection, or at 30, 90, and 180 days after the infection.

References

1. Nowak MA, May RM, Anderson RM (1990) The evolutionary dynamics of HIV-1 quasispecies and the development of immunodeficiency disease. AIDS 4: 1095–1103.

2. Althaus CL, De Boer RJ (2008) Dynamics of immune escape during HIV/SIV infection. PLoS Comput Biol 4: e1000103.

3. Alizon S, van Baalen M (2008) Multiple infections, immune dynamics and virulence evolution. Am Nat 172: E150–E158.

4. Press W, Teukolsky S, Vetterling W, Flannery B (1992) Numerical recipes in C: the art of scientific computing. New-York, USA: Cambridge University Press. URL http://www.slac.stanford.edu/spires/find/books?irn=212237.

5. Abrahams MR, Anderson JA, Giorgi EE, Seoighe C, Mlisana K, et al. (2009) Quantitating the multiplicity of infection with human immunodeficiency virus type 1 subtype C reveals a non-poisson distribution of transmitted variants. J Virol 83: 3556–3567.

6. Anderson RM, May RM (1991) Infectious Diseases of Humans. Dynamics and Control. Oxford: Oxford University Press.

7. Hollingsworth TD, Anderson RM, Fraser C (2008) HIV-1 transmission, by stage of infection. J Infect Dis 198: 687–693.
