# Supplementary material for: The Evolutionary Dynamics of a Rapidly Mutating Virus within and between Hosts: The Case of Hepatitis C Virus
Source: PLoS Comput Biol. 2009 Nov 13;5(11):e1000565. doi: 10.1371/journal.pcbi.1000565 (PMC2768904; doi:10.1371/journal.pcbi.1000565)
Supplement: Text S2 — Additional results. We discuss the effect on life-history traits of variations of the initial value of immune cells and infected cells, of the probability of virulence and of the effect of a saturation term for the description of lymphocyte proliferation rate function. (0.08 MB PDF) [file pcbi.1000565.s004.pdf]

Text S2: *Supplementary results*

B.1 Effect of the initial viral load and of the T-cell precursor frequency on life-history traits

We investigate the effect of varying the precursor frequencies of immune cells y_0 before infection starts. We simulate the following values of y_0, $10^0, 10^1, 10^2, 10^3, 10^4, 10^5$ (default value is 10^2). Increasing the initial number of lymphocytes has a similar effect to what is observed when increasing the killing rate σ_{max}: both the fitness and the duration of the infection increase, thus suggesting that viruses benefit from an increase in the recruitment of immune cells (see also the results in the main text on varying the maximum killing rate σ_{max}).

The initial number of infected cells x_0 is varied by five orders of magnitude (1, 10, 10^2, 10^3, 10^4, 10^5). We do not observe significant variations in life-history traits (Supplementary Table 3). This result is likely due to the fact that in our default model, there is no limitation to the number of uninfected cells.

B.2 Effect of virulence rate (host death) on life-history traits

We introduce a virulence event that is modelled stochastically in a similar manner as done for the transmission dynamics (see Supporting Text S1). We performed simulations varying the rate at which hosts die. This is done by varying the parameter η. This analysis shows, unsurprisingly, that increasing virulence decreases most of the viral traits (Table 3). The final number of infected cells increases because infections are less likely to be ended by host recovery. The increase of virulence also slightly reduces the optimal replication rate (figure not shown).

B.3 Effect on life-history traits of the number of replication rates, R_{max}, of the number of antigenic values A_{max}, and of the number of clones m

Viral evolution is limited within a finite space of traits values. In the default simulations we assume a fixed number of replication rates $R_{max} = 100$ that virus explores during its evolution. We simulate default values (see Table 1) with several values of R_{max} (5, 10, 20, 50, 100, 500, 1000). Variations on the number of replication rates affect viral fitness only when R_{max} is small (< 50). In this case, viral diversity is zero and virus does not evolve into a quasi-species distribution (not shown). This observation is explained by the fact that when the space of replication rates is very limited, viruses are limited in generating new strains that can escape the immune response. For higher values of R_{max} we do not observe an effect on viral evolution. This suggests that the number of replication rate values, as long as it is not very small, does not affect viral evolution.

We then vary the number of antigen values available (A_{max}), using the default values in Table 1 for other parameters. Results show that viral evolution is not affected by the size of the antigenic space. This is a consequence of our assumption that antigen values and lymphocytes receptors are uniformly distributed on the circle $[0, 2\pi]$. In this scenario, viruses evolve towards strains harboring antigenic values that minimise the overlap with lymphocytes receptors. Thus, the antigen values tend to be uniformly distributed (see also [1]).

Finally, we investigate the effect of varying the number of lymphocytes clones, m, on life history traits. When m increases, the fitness of the virus and the duration of the infection both increase for high values of the initial replication rate (φ_0). Viral diversity also increases. These seemingly counter-intuitive results arise because of the non linearity of the killing term in equation 1a of the main text. In fact increasing the killing rate –which for instance occurs when the number of lymphocytes clones or their precursor frequencies increase– results in a decrease in the number of infected cells early in the infection. As a consequence, this decrease 'slows down' the activation of the immune response.

B.4 Effect of saturation function on the killing rate and on the proliferation rate of immune cells

We analyse the model assuming a Michaelis-Menten type of function for the proliferation rate of immune cells. The reason for this analysis is that limiting immune proliferation by resources can interfere with the result of the model that fitness of the infection has a non linear behaviour with the initial replication rate of the infecting strain. We therefore considered the following proliferation rate, which is classically used in resources-limitation models [2, 3], that describes the limitation of cell proliferation by inter-clonal competition for resources (antigens):

$$\sum_i c_{ij} \frac{\phi_i \, x_i}{\theta_{p_i} + \sum_l c_{i,l} \, y_l}. \tag{3}$$

Here θ_{p_j} is the number of immune cells that corresponds to half of the maximum proliferation rate for clone j with number of cells y_j. In order to account for different T-cell affinities towards different antigens, we uniformly distribute θ_{p_i} between 0 and θ_{max}. In this model, for values of the immune response such that $\sum c_{i,l} y_l \ll \theta_p$, proliferation follows a mass-action kinetics with rate $\frac{c}{\theta_p} \sum c_{i,l} y_l$; while for high values of immune response, $\sum c_{i,l} y_l \gg \theta_p$, competition between immune cells increases and proliferation rate is at maximum rate, c_{max}.

We simulate infection dynamics for several values of θ_{max} and c_{max}. Low values of θ_{max} correspond to the case of increased competition between immune cells and therefore the killing rate saturates for lower values of immune cells. We run simulations using the same range of values for ϕ_0 as in the default case, and with values of $\theta_{max} = 1$, 10, 10^6, 10^{10}, and 10^{12}. We also simulate the same range of values for c_{max} as given in Figure 3 of the main text. For high values of θ_{max} ($\theta_{max} > 10^6$) we observe a fast growth of virus with a limited immune response. Because we assume that a very high viral load or a very high level of immune response cause death of the host (see Supporting Text S1), we find that all the runs lead to host death. This occurs for all simulated values for the maximum proliferation rate c_{max}. For simulations where $\theta_{max} \leq 10^6$ we again observe a maximum of the fitness as a function of the initial replication rate. Most importantly, we observe a decrease in the overall fitness values with the increase of the maximum proliferation rate c_{max}. This increase in fitness with maximum proliferation rate is in agreement with what we observe in the case without saturation in the immune proliferation function.

Table 3: **Effects of the model parameters on the infection life-history traits.** We performed linear fits based on a multivariate linear model on the mean values of the traits as a function of 12 factors: 9 model parameters (q, μ, $\sigma_{\max}$, $c_{\max}$, τ_1, τ_2, η, w, ρ), the initial replication rate φ_0, the initial precursor frequency of immune cells y_0, and the initial number of infected cells x_0. Further details about the statistical tests are available in Supplementary Text S1. Significance level $\alpha = 0.01$. Significance codes: '***': 0.001; '**': 0.01; '*': 0.05; ' ': non significant.

Parameter / Traits	Initial number of infected cells (x_0)	Initial number of lymphocytes (y_0)	Virulence (η)
Infection fitness	Increase	Increase	Decrease **
Duration of the infection (Log)	Increase *	Increase	Decrease ***
Mean replication rate of transmitted strains	Increase	Increase ***	Decrease ***
Final viral diversity	Decrease	Increase	Increase **
Final number of lymphocytes (Log)	Increase	Decrease	Decrease
Final number of infected cells (Log)	Decrease	Increase	Increase

References

1. Alizon S, van Baalen M (2008) Multiple infections, immune dynamics and virulence evolution. Am Nat 172: E150–E158.

2. De Boer RJ, Perelson AS (1994) T cell repertoires and competitive exclusion. J Theor Biol 169: 375–390.

3. Althaus CL, De Boer RJ (2008) Dynamics of immune escape during HIV/SIV infection. PLoS Comput Biol 4: e1000103.
